# Investigations of potential non-amino acid SNAT2 inhibitors

**DOI:** 10.3389/fphar.2023.1302445

**Published:** 2024-01-04

**Authors:** Sebastian Jakobsen, Emilie Fynbo Petersen, Carsten Uhd Nielsen

**Affiliations:** Department of Physics, Chemistry and Pharmacy, University of Southern Denmark, Odense, Denmark

**Keywords:** SNAT2, amino acid transport, cancer, PC-3 cells, inhibitor

## Abstract

The sodium-coupled neutral amino acid transporter 2 (SNAT2, SLC38A2) has been implicated in cancer for its ability to supply cancer cells with glutamine and sarcosine. A recent high-throughput screen published by Gauthier-Coles et al. identified the non-amino acid 3-(N-methyl (4-methylphenyl)sulfonamido)-N-(2-trifluoromethylbenzyl)thiophene-2-carboxamide (MMTC or 57E) as a potent and selective SNAT2 inhibitor. Here we have investigated the ability of MMTC and four other compounds selected from the screen by Gauthier-Coles et al. to decrease ^3^H-Gly uptake in hyperosmotically treated human prostate cancer PC-3 cells. In these cells, SNAT2 is highly upregulated when the cells are hyperosmotically stressed for 24 h and is the primary contributor to glycine uptake. The five compounds were investigated at concentrations of 1–50 µM based on their equilibrium solubility. At 37°C the equilibrium solubility in HEPES buffered HBSS at pH 7.4 was measured to be 24.9 (53B), 56.1 (54F), 13.3 (55B), and 27.5 (57B) µM, respectively. The equilibrium solubility of MMTC was below the detection limit of the HPLC-UV method, thus less than 1.8 µM. However, a kinetic solubility of approximately 2.5–10 µM could be achieved during the course of the uptake study. In contrast to the previous publication, MMTC showed no inhibition of SNAT2-mediated ^3^H-Gly uptake in PC-3 cells at a concentration of 1 or 5 μM, despite a published IC_50_ of 0.8 µM. Similarly, 53B, 55B, and 57B showed no inhibition at soluble conditions, whereas 54F showed approximately 20% inhibition at 50 µM. In our experimental setup, the investigated compounds showed limited potential as SNAT2 inhibitors.

## 1 Introduction

In a recent publication, Gauthier-Coles et al. Gauthier-Coles et al. (2022) proposed a number of high-affinity inhibitors for the glutamine-transporting sodium-coupled neutral amino acid transporter 2 (SNAT2). Since few SNAT2 inhibitors have been described, this added important knowledge to a field in need of specific and selective inhibitors for, e.g., structural biology applications such as inhibitors for crystallization and for cancer cell targeting. It has long been known that cancer cell cultures need glutamine supplementation to survive (Eagle, 1955). It was later shown that this “glutamine addiction” was mainly due to how cancer cells rewire their metabolism and utilize the glutaminolysis pathway that requires glutamine (DeBerardinis et al., 2007; Wise et al., 2008). Potentially, this makes glutamine metabolism and distribution an interesting target in the search for cancer therapeutics. Amino acid transporters within the solute carrier (SLC) family, that facilitate the uptake of glutamine, have thus gained interest as potential targets in cancer. One well-studied glutamine transporter is ASCT2 (SLC1A5), where several inhibitors have been discovered (Esslinger et al., 2005; Garibsingh et al., 2021; Ndaru et al., 2022). However, there are some drawbacks when targeting ASCT2 in cancer therapy. First of all, several of the discovered ASCT2 inhibitors have been shown to be non-selective and inhibited other amino acid transporters (Bröer et al., 2016; Bröer et al., 2018). Secondly, ASCT2 is an amino-acid exchanger and is thus not able to drive net glutamine uptake. Sodium-coupled amino acid transporters like SNAT1 (SLC38A1) and SNAT2 (SLC38A2) are capable of increasing net glutamine uptake and have been shown to compensate for ASCT2 deficiency in cancer cells (Bröer et al., 2016; Bröer et al., 2019). However, SLC38 transporters are still in their infancy when it comes to discovering potent inhibitors. SNAT2 facilitates the sodium-dependent uptake of various neutral amino acids with a preference for small-medium sized amino acids like alanine and glutamine (Sugawara et al., 2000). It is characteristically known to be induced by amino acid starvation and osmotic stress (Gazzola et al., 2001; Kashiwagi et al., 2009). Along with its ability to supply cancer cells with glutamine, SNAT2 has also been implicated to play a role in the uptake of the oncometabolite sarcosine in prostate cancer cells (Nielsen et al., 2022). It has been proposed that SNAT2 is able to signal to the cell growth regulator mTORC1 (mammalian target of rapamycin complex 1) through a transceptor function (Pinilla et al., 2011; Chiu et al., 2012; Fairweather et al., 2021). Despite these implications in cancer, only a few inhibitors have been discovered. Most of the known inhibitors are compounds first discovered to inhibit ASCT2 such as benzylserine, GPNA, and V-9302, and are thus not specific inhibitors (Bröer et al., 2016; Bröer et al., 2018). However, recently Gauthier-Coles and others reported a potent and selective SNAT2 inhibitor called MMTC (IC_50_ = 0.8 µM). The inhibitor was discovered in a high-throughput-screen using the FLIPR membrane potential assay and further characterized through radiolabeled uptake studies in various cell lines (Gauthier-Coles et al., 2022). Since we are looking for the structural determinants of SNAT2 binding in order to identify inhibitors hereof, we investigated a small selection of the hits suggested by Gauthier-Coles et al. in PC-3 cells, which when cultured in hyperosmotic medium highly upregulates SNAT2 expression (Nielsen et al., 2022). We thus used a cell model different from Gauthier-Coles et al. to see if we could replicate the same inhibitory potency seen for MMTC as well as test four other compounds identified by the screen by Gauthier-Coles et al. Gauthier-Coles et al. (2022). This was originally intended for internal use only, yet as we obtained different results than the original paper, we felt obligated to share our findings with the amino acid research field through this Brief Research Report.

## 2 Methods

### 2.1 Materials

[2–^3^H]-Glycine (45.2 Ci/mmol), Ultima GoldTM scintillation fluid, and scintillation vials (6 mL, Pony VialTM) were from Perkin Elmer (Waltham, MA, United States). Dulbecco’s Modified Eagle Medium/Nutrient Mixture F-12 (DMEM/F12), penicillin/streptomycin (100x), L-glutamine (200 mM), sodium pyruvate (100 mM), L-ascorbic acid, phosphate-buffered saline, trypsin-EDTA (10x) all suitable for cell culture were from Sigma Aldrich (Merck KGaA, Darmstadt, Germany). Fetal Bovine Serum (FBS) for cell culture was from Biowest (Nuaillé, France). Hanks Balanced Salt Solution (HBSS) 10x was from Gibco through Thermo Fischer Scientific (Waltham, MA, United States). Sodium bicarbonate solution (7.5%), 4-(2-hydroxyethyl)-1-piperazineethanesulfonic acid (HEPES), D-(+)-raffinose pentahydrate, L-glutamine, L-arginine hydrochloride and dimethyl sulfoxide (DMSO) were from Sigma Aldrich (Merck KGaA, Darmstadt, Germany). Trifluoroacetic acid was from Thermo Fisher Scientific (Waltham, MA, United States). Acetonitrile (HPLC graded) was from VWR Chemicals (Radnor, PA, United States). Ultra-pure water was obtained from an in-house Milli Q water purification system (Merck Millipore, Burlington, MA, United States). Compounds MMTC (57E), 53B, 54F, 55B, and 57B were from ChemDiv (San Diego, CA, United States), which is the vendor used in (Gauthier-Coles et al., 2022). Hence, we have kept the names similar to those used in (Gauthier-Coles et al., 2022). The ChemDiv ID of MMTC, 53B, 54F, 55B, and 57B is L876–0122, C169–020, M071–0231, L557-0109, and D314-0274, respectively. The five compounds were run through a PAINS filter (https://www.cbligand.org/PAINS/login.php) (Baell and Holloway, 2010) and all compounds passed the filter.

### 2.2 Cell culture

PC-3 cells (ECACC 90,112,714) were obtained from the European Collection of Authenticated Cell Cultures (ECACC; UK Health Security Agency, Salisbury, United Kingdom) and were received in passage 31. PC-3 cells were maintained in DMEM/F12 supplemented with 10% fetal bovine serum (FBS), streptomycin (0.1 mg ⋅ mL^−1^), penicillin (100 U ⋅ mL^−1^), L-glutamine (2 mM), sodium pyruvate (2 mM), and L-ascorbic acid (20 μg ⋅ mL^−1^). The cells were kept incubated at 37°C in an atmosphere of 5% CO_2_ and 94%–97% relative humidity and the culture medium was changed every 2–3 days. For hyperosmotic stimulation, PC-3 cells were incubated in hyperosmotic media for 24 h before the uptake experiment. The hyperosmotic media was made by supplementing normal isoosmotic culture medium with 200 mM raffinose to reach an osmolality of approximately 500 mOsm ⋅ kg^−1^. For uptake studies, the PC-3 cells were seeded in 24-well plates (area of 1.9 cm^2^) at a density of 1.5 ⋅ 10^5^ cells ⋅ cm^−2^ 2 days before the experiment. Experiments were performed using PC-3 cells in passages 2–15 after thawing.

### 2.3 Radiolabeled uptake studies

Hyperosmotically treated PC-3 cells were used to study the uptake of radiolabeled ^3^H-Gly. Solutions were prepared using Hanks Balanced Salt Solution (HBSS), which consisted of the following (mM): CaCl_2_, 1.26; MgCl_2_, 0.49; MgSO_4_, 0.41; KCl, 5.33; KH_2_PO_4_, 0.44; NaCl, 138; Na_2_HPO_4_, 0.34; D-glucose, 5.56; NaHCO_3_, 4.17. HBSS was supplemented with 10 mM HEPES and adjusted to pH 7.4 ± 0.01 (HBSS*) with 0.1–5.0 mM NaOH. Donor solutions contained 0.5 μCi/mL of ^3^H-glycine (11.1 nM). MMTC was also investigated at a substrate concentration of 100 µM Gly, which was achieved by the addition of unlabeled Gly to donor solutions containing 0.5 μCi/mL of ^3^H-Gly (11.1 nM). The results can be seen in Supplementary Figure S1. Before starting the uptake experiment the media was aspirated, and the cells were preincubated in HBSS* for 15 min at 37°C and 220 rpm. To initiate the uptake study, the buffer was removed and 300 µL of prewarmed (37°C) donor solution was added to the cells and incubated at 37°C. During incubation, the cells were circularly rotated at 220 rpm using a Talboys incubating microplate shaker (Troemner, Thorofare, NJ, United States). After 5 min, the uptake experiment was terminated by removing the buffer solutions by vacuum suction, and the cells were washed thrice with 500 µL ice-cold HBSS. The uptake time of 5 min ensures initial rate conditions as validated using ^14^C-Sar uptake studies, see Supplementary Figure S2. The cells were then detached by applying 200 µL of 0.1% Triton-X 100 for at least 25 min and cells were then transferred to scintillation vials along with 2 mL of Ultima Gold scintillation fluid. The vials were vortexed before being analyzed by a liquid scintillation counter, which was a TriCarb 4910 TR from PerkinElmer (Waltham, MA, United States).

### 2.4 HPLC-UV quantification of MMTC, 53B, 54F, 55B and 57B

Quantification of compounds was performed using a Waters 2695 HPLC system (Waters Corporation, MA, United States) connected to a Waters 2487 Dual λ Absorbance Detector (Waters Corporation, MA, United States). The mobile phases (MP) consisted of ultra-pure water containing 0.1% trifluoroacetic acid (MP A) and acetonitrile containing 0.1% trifluoroacetic acid (MP B). A reversed-phase column (Nova-Pak^®^ C18, 4 μm, 3.9 × 150 mm, Waters, Milford, MA, United States) was applied and maintained at 22°C. The flow was constant at 0.6 mL ⋅ min^−1^ with an isocratic elution of 40% MP A and 60% MP B for 53B, 55B, and 57B, an isocratic elution of 30% MP A and 70% MP B with a constant flow of 0.8 mL ⋅ min^−1^ for MMTC, and an isocratic elution of 60% MP A and 40% MP B with a constant flow of 0.6 mL ⋅ min^−1^ for 55B. Samples of 20 μL, maintained at 25°C, were injected and all five compounds were measured at 270 nm. MMTC, 53B, 54F, 55B, and 57B had retention times of 4.00, 2.47, 5.14, 2.92, and 4.00 min, respectively. Calibration curves in the range of 5–50 µM of all five compounds were firstly prepared in HBSS* buffer, however in this concentration range linearity was not observed (likely due to solubility issues). Thus, the calibration curves were prepared in their respective mobile phases resulting in linear calibration curves, which may be due to higher solubility in the organic mobile phase. The calibration curves ranged from 5 to 30 µM for MMTC and 53B and from 7.5 to 30 µM for 54F, 55B, and 57B. Linear regression of peak height as a function of concentration was analyzed using GraphPad Prism 9.4.1. The calibration curve of MMTC, 53B, 54F, 55B, and 57B had the linear regression equations of y = 1253.2x + 219.3 with *R* ^2^ = 0.9998, y = 2774.2x + 98.6 with *R* ^2^ = 0.9996, y = 821.9x + 23.0 with *R* ^2^ = 0.9999, y = 711.9x + 633.6 with *R* ^2^ = 0.9974, and y = 636.1x - 1166 with *R* ^2^ = 0.9961, respectively.

### 2.5 Solubility determination

The equilibrium solubility was measured by initially adding 30 µL of 40 mM stock solutions in 100% DMSO of MMTC, 53B, and 54F, 30 µL of a 50 mM stock solution in 100% DMSO of 55B, and 60 µL of a 20 mM stock solution in 100% DMSO of 57B to five separate 5 mL plastic vials and adding 3 mL HBSS* buffer, which resulted in heavy precipitation. The 5 mL vials were then left on a rotator covered in aluminum foil at 37°C. After 72 h rotation, the solutions were equally distributed into three 1.5 mL microtubes, to do triplicate measurements, and centrifuged at 10,000 g at room temperature for 10 min. The supernatant was removed and for 53B, 54F, and 57B the supernatant was diluted 1:2 with their respective mobile phases. For MMTC and 55B, the supernatant was diluted 2:1 with their respective mobile phase. The diluted samples were analyzed by HPLC-UV as described in Section 2.4. Due to the absence of peaks in the chromatograms of MMTC and 55B new solubility studies were prepared in the same concentrations, same solvents, and same procedure as described above of the two compounds, with a shorter duration of 21 h and were studied both at room temperature and 37°C. The chromatograms of MMTC still did not show a peak corresponding to MMTC, hence another solubility study was performed in glassware and the procedure was followed as described before. Furthermore, a kinetic solubility study was performed for MMTC, where a 1000 µM MMTC stock solution in 100% DMSO was diluted 100-times in HBSS* to a final concentration of 10 µM and placed in a 37°C water bath and 100 µL was withdrawn and diluted 1:2 with the respected mobile phase at time points 0, 15, 30 and 60 min and analyzed immediately by HPLC-UV as described in Section 2.4.

### 2.6 Mass spectrometry

The five tested compounds were analyzed using mass spectrometry (MS) to confirm the identity of the compounds. The compounds were analyzed using a micrOTOF electrospray ionization quadrupole time-of-flight MS (ESI-QTOF-MS) from Bruker Daltonics (Bremen, Germany) in positive mode. The compounds were prepared by diluting the DMSO stocks with acetonitrile to a final concentration of 0.04 mg ⋅ mL^-1^. The molecular weights of the compounds were confirmed using mass spectrometry (Table 1; Supplementary Figure S3)

**TABLE 1:** Equilibrium solubilities in HBSS* at 37°C and compound mass determined through mass spectrometry of MMTC, 53B, 54F, 55B, and 57B. Solubilities are shown as means ± SD of triplicate determination (*N* = 3). The exact masses of the compound adducts are shown along with the determined mass.

Compound name	MMTC	53B	54F	55B	57B
**Equilibrium** s**olubility, [µM]**	<1.8	24.9 ± 0.8	56.1 ± 0.5	13.2 ± 1.6	27.5 ± 1.0
**Mass spectrometry adduct**	Na^+^	H^+^	H^+^	H^+^	H^+^
**Exact mass, Da**	491.0681	386.1975	411.0501	421.1062	444.1619
**Determined mass, Da**	491.0701	386.1961	411.0516	421.1076	444.1618

### 2.7 Data analysis

^3^H-Gly uptake values were normalized to the control uptake of each experiment, but without subtracting any background uptake.

For the quantification of MMTC, 53B, 54F, 55B, and 57B a linear regression was made of each of the calibration curves, where the known concentrations were plotted as a function of the peak height. The calibration curves were used to determine the concentration, taking sample dilution into account, of MMTC, 53B, 54F, 55B, and 57B in the solubility studies, with the following formula:

$$c_{x}=\frac{PH-y_{intercept}}{slope}$$

Where c_x_ is the concentration of the unknown sample, the PH is the peak height from the HPLC-UV chromatogram of the measured solubility sample, and the y_intercept_ and slope are derived from the calibration curve.

### 2.8 Statistical analysis

Statistical differences were tested in GraphPad Prism 9.4.1 using one-way ANOVA followed by Dunnett’s multiple comparison test. The significance level used was *p* < 0.05 (*). Data are represented as means ± SEM from independent cell passages (n) unless otherwise stated.

## 3 Results

**FIGURE 1:**
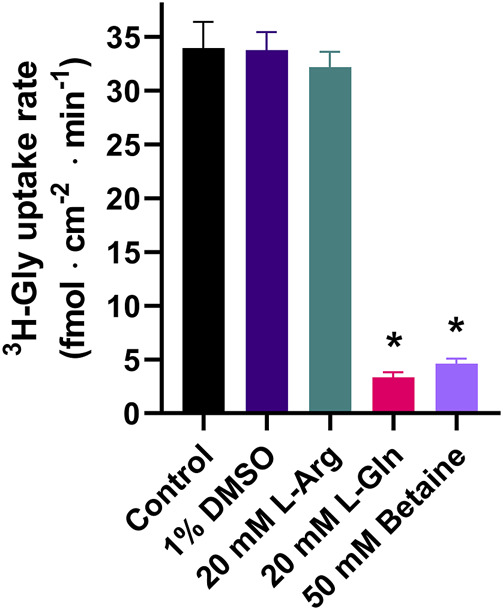
^3^H-Gly uptake in hyperosmotically treated PC-3 cells in the absence or presence of 1% DMSO, 20 mM L-Arg, 20 mM L-Gln, or 50 mM betaine. Cells were treated with hyperosmotic medium supplemented with 200 mM raffinose 24 h before experiments. All experiments were performed using 10 mM HEPES buffer in HBSS, pH 7.4. The cells were exposed to 0.5 µCi ⋅ mL^−1^ ^3^H-Gly (11.1 nM) for 5 min at 37°C. Values are reported as means ± SEM for *n* = 3 independent cell passages. Statistically significant differences from the control detected by one-way ANOVA are shown (*: *p* < 0.05).

^3^H-glycine (^3^H-Gly) uptake in PC-3 cells following 24 h of hyperosmotic stimulation was used as an *in vitro* model to study SNAT2-mediated transport. The resulting 5 min uptake in the presence or absence of 1% DMSO, 20 mM L-Arg (a known non-substrate of SNAT2), 20 mM L-Gln (a known SNAT2 substrate), or 50 mM betaine [a SNAT2 substrate that is used to discriminate between SNAT1 and SNAT2 (Nishimura et al., 2014)] can be seen in Figure 1. 20 mM L-Gln represents a fully inhibited system with an uptake of about 9% of the control. The SNAT2 selective substrate betaine inhibits the uptake by around 86% at 50 mM, indicating that the Gly uptake is primarily mediated by SNAT2. 20 mM L-Arg and 1% DMSO did not affect the uptake significantly and DMSO could thus be used to help dissolve poorly soluble compounds as long as the final concentration applied to PC-3 cells did not exceed 1%.

**FIGURE 2:**
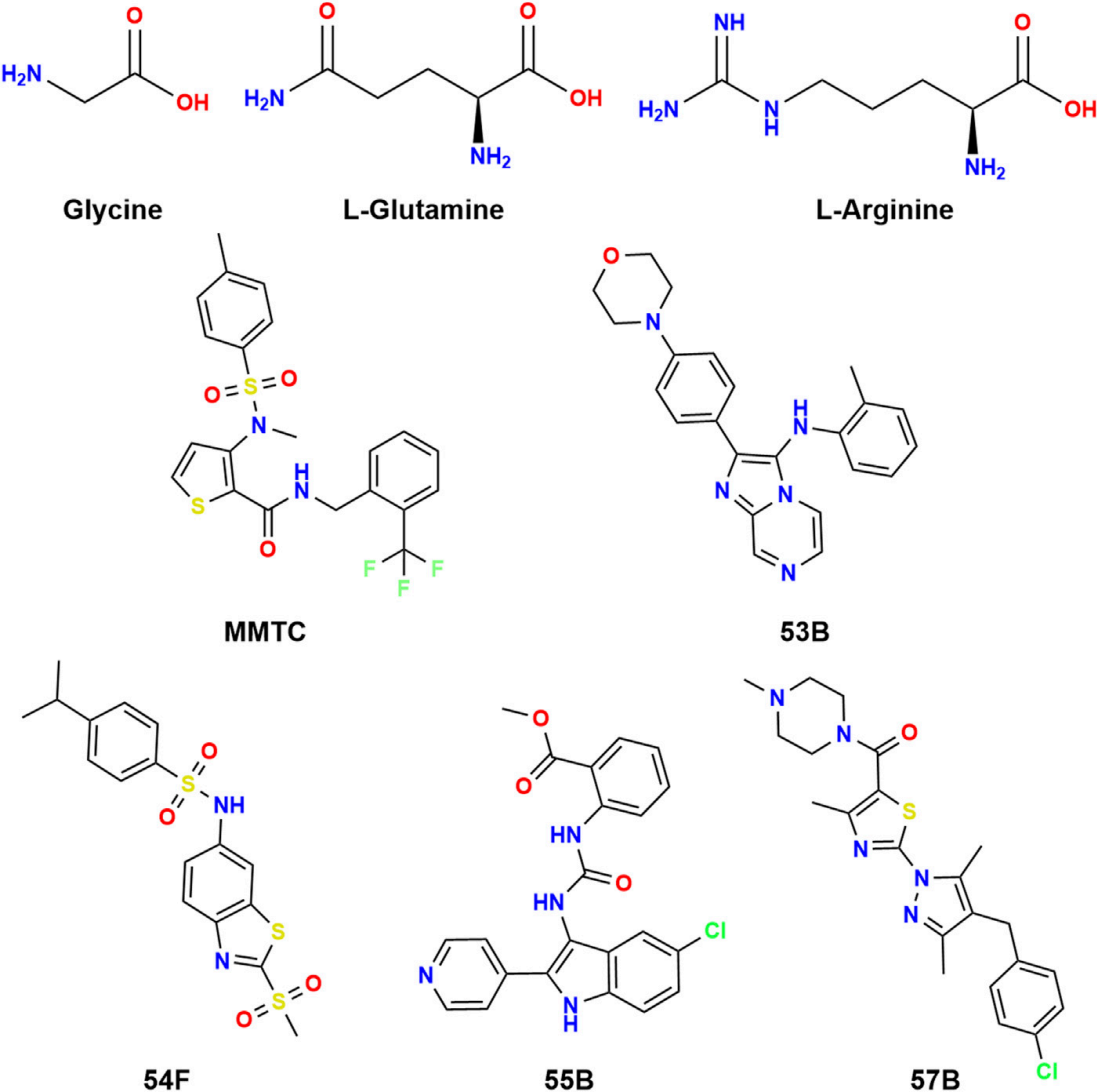
Chemical structures of glycine, L-glutamine, and L-arginine along with MMTC, 53B, 54F, 55B, and 57B.

MMTC, 53B, 54F, 55B, and 57B identified as hits in a screen for SNAT2 inhibitors (Gauthier-Coles et al., 2022) were tested for their ability to inhibit SNAT2-mediated ^3^H-Gly uptake in PC-3 cells. Interestingly, none of these five compounds resemble amino acids (Figure 2).

**FIGURE 3:**
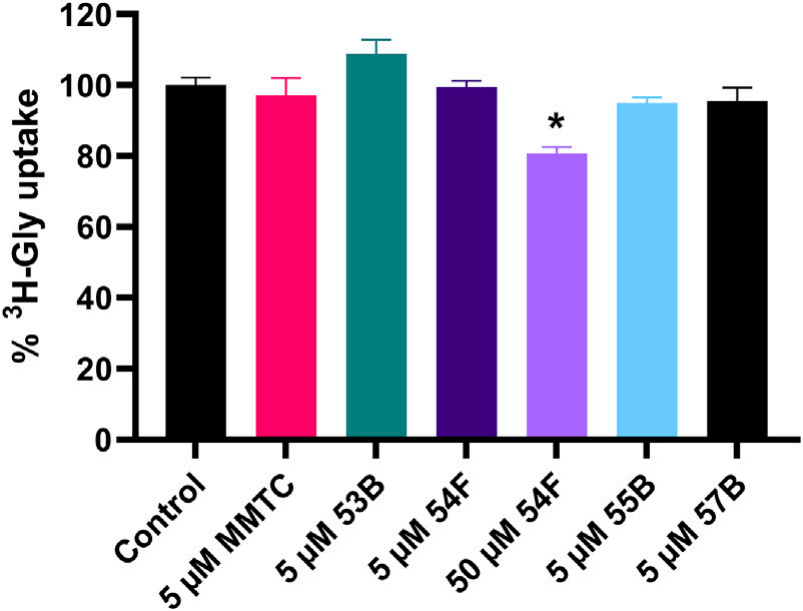
Normalized ^3^H-Gly uptake in hyperosmotically treated PC-3 cells in the presence or absence of 5 or 50 µM of the compounds MMTC, 53B, 54F, 55B, and 57B. PC-3 cells were treated with hyperosmotic media supplemented with 200 mM raffinose 24 h before experiments. All experiments were performed using 10 mM HEPES buffer in HBSS, pH 7.4 and the final inhibitor solutions contained 1% DMSO. The cells were exposed to 0.5 µCi ⋅ mL^−1 3^H-Gly (11.1 nM) for 5 min and at 37°C. Values are reported as means ± SEM for three independent cell passages (*n* = 3). Statistically significant differences from the control detected by one-way ANOVA are shown (*: *p* < 0.05).

All compounds were initially dissolved in DMSO, then diluted in HBSS* buffer, and investigated using PC-3 cells at a final concentration of 1% DMSO. However, because of apparent solubility issues the equilibrium solubilities of the compounds in HBSS* buffer when diluted from DMSO stocks were determined using HPLC-UV (Table 1). MMTC could not be detected in the solubility sample after 72 h of incubation as well as after 21 h. MMTC particles were visually attached to the plastic vial, hence mobile phase was used to wash the plastic vial, and the sample was analyzed by HPLC-UV and showed that MMTC was present in high amounts. Therefore, another solubility study was conducted in glassware, however, no peaks in the chromatogram could be detected indicating that MMTC was not in the HBSS* buffer but precipitated as clumped particles. Based on these observations, a kinetic solubility study was conducted by diluting MMTC from a DMSO stock solution with HBSS* buffer, corresponding to 10 μM MMTC, and following it over a period of 60 min. The results indicated that the MMTC concentration decreased from 10 µM to approximately 2.5 µM within 15 min and remained stable for at least 45 min, indicating that an oversaturation occurs when MMTC in DMSO is diluted into HBSS* buffer. Because the equilibrium solubility of MMTC could not be determined, it was set to less than 1.8 µM as that was the lowest concentration detectable by HPLC-UV. Inhibition of ^3^H-Gly uptake in PC-3 cells by the five compounds at appropriate concentrations is seen in Figure 3. MMTC was tested at 5 µM prior to the solubility studies but was included given that a transient supersaturation could possibly be achieved. It is seen that in PC-3 cells none of the investigated compounds significantly decrease the uptake of ^3^H-Gly at 5 µM. 54F was soluble at 50 µM and able to significantly inhibit ^3^H-Gly uptake by around 20% when compared to the control. In general, the compounds did not show any notable inhibition of SNAT2 within their soluble concentration range.

## 4 Discussion

In a recent publication, Gauthier-Coles et al. Gauthier-Coles et al. (2022) reported the finding of several potential SNAT2 inhibitors with IC_50_ values of 1–10 µM measured using the FLIPR membrane potential assay. One of these compounds, named MMTC, was subsequently shown to inhibit ^3^H-Pro uptake in amino acid starved SKOV3 cells with an apparent inhibitory potency (IC_50_) of 0.8 µM (Gauthier-Coles et al., 2022). Given the interest in SNAT2, inhibitors are especially important for investigating cancer cell targeting, but also as tools to capture the SNAT2 protein in different conformations for structural biology, e.g., CryoEM. We therefore intended to confirm the findings from (Gauthier-Coles et al., 2022) to support our own effort in building and validating an accurate homology model of SNAT2. Five compounds from Gauthier-Coles et al. were therefore selected and their identities were confirmed by Mass Spectrometry. The *in vitro* system used was inhibition studies of ^3^H-Gly uptake in hyperosmotically stressed PC-3 cells. We have recently shown that hyperosmotically stressed PC-3 cells upregulated SNAT2 expression leading to a 9-fold increase in radiolabeled Gly uptake at trace amounts of isotope (Nielsen et al., 2022). Moreover, the maximal uptake capacity (V_max_) of radiolabeled Gly increased 5 times (Nielsen et al., 2022). Importantly, through *SNAT2 siRNA* knock-down we have confirmed that Gly uptake in hyperosmotically stressed PC-3 cells was primarily mediated by SNAT2 (Nielsen et al., 2022). This finding is supported by data in Figure 1 showing that 50 mM betaine, similar to 20 mM Gln, almost completely inhibits Gly uptake, suggesting that SNAT2 and not SNAT1 is mainly responsible for the uptake of Gly in PC-3 cells, given that betaine discriminates between SNAT2 and SNAT1 (Nishimura et al., 2014). In hyperosmotically stressed PC-3 cells, we found that the recently proposed novel SNAT2 inhibitor MMTC did not inhibit ^3^H-Gly uptake. MMTC had limited aqueous solubility and its equilibrium solubility appeared to be too low to be detected through HPLC-UV. Initially, after dilution of MMTC from DMSO to HBSS* buffer, a transient supersaturation could be maintained and MMTC was tested at 5 µM and showed no inhibition of 11.1 nM ^3^H-Gly uptake in PC-3 cells. This is in contrast to the reported IC_50_ value of 0.8 µM for MMTC as determined in a radiolabeled Pro uptake study in amino acid starved SKOV3 cells using 100 µM ^3^H-Pro (Gauthier-Coles et al., 2022). To investigate if the difference in inhibition was due to the difference in substrate concentration, we measured the inhibition at 100 µM Gly (Supplementary Figure S1) and still found that 1 µM MMTC does not inhibit Gly uptake in PC-3 cells. In PC-3 cells, Gly and Pro uptake have quite comparable K_m_ values of 0.75 ± 0.16 and 0.78 ± 0.10 mM (Nielsen et al., 2022), respectively, indicating that the choice of substrate should kinetically not be the reason for the lack of inhibition by MMTC. Since the mode of inhibition was not determined by Gauthier-Coles et al. nor us, we can only hypothesize on why we see a difference. In the case of competitive inhibition, our apparent affinity would be close to the true K_i_ values, given that our substrate concentration is several times lower than the K_m_ value of Gly (11.1 nM vs. 0.75 mM). Gauthier-Coles et al. used 100 µM substrate concentrations of Pro and assuming a similar K_m_ of Pro in SKOV3 cells as to what has been determined in PC-3 cells, the K_i_ is calculated to be 0.7 µM through the Cheng-Prusoff equation (Cheng and Prusoff, 1973) based on their 0.8 µM IC_50_ value. So, this would not explain why we do not see any significant inhibition at the concentration we have used. Non-competitive inhibition would mean that substrate concentration would not influence the affinity, so this would also not explain the differences observed. Assuming that it is un-competitive inhibition could be an explanation. Because we use a much lower substrate concentration our apparent IC_50_ value would be several folds higher than the actual K_i_. As mentioned above we conducted an initial experiment with a higher Gly substrate concentration and did not observe any inhibition from MMTC. Yet, the cause of the discrepancy in MMTC potency could be due to the underlying inhibitory mechanism behind the potential interaction. That inhibition values in different studies differ to a large degree is seen before. Discrepancies between groups in terms of amino acid transporter inhibitors have been seen in the case of the ASCT2 inhibitors Compound 12 and V-9302. The two compounds were first discovered by exploring a novel 2-Amino-4-bis(aryloxybenzyl)aminobutanoic acid scaffold and testing ASCT2 inhibition in HEK293 cells where IC_50_ values in the 7–10 µM range were shown (Schulte et al., 2016; Schulte et al., 2018). V-9302 was further characterized *in vitro* and *in vivo* where it showed anticancer properties (Schulte et al., 2018). However, Bröer and others could not replicate the inhibition of these compounds against ASCT2, when expressed in *Xenopus laevis* oocytes, but instead saw inhibition of LAT1 and SNAT2, which possibly could explain the observed anticancer effects (Bröer et al., 2018). This might point to difficulty in finding suitable cell systems to study amino acid transporters, given how abundant they are (at least 60 different SLCs in the human genome) and their overlap in the amino acids they transport. As Gauthier-Coles et al. clearly illustrate in their paper, they are working with mixed systems, where multiple amino acid transporters contribute to the observed uptake (Gauthier-Coles et al., 2022). This, of course, makes it difficult to ascertain which transporter is inhibited, but their vast number of experiments makes a compelling argument that MMTC is a SNAT2 inhibitor, even though we cannot replicate a submicromolar potency as shown by the data presented here.

In conclusion, the novel proposed SNAT2 inhibitor MMTC did not inhibit ^3^H-Gly uptake in hyperosmotically treated PC-3 cells, whereas 54F was the only of the five compounds investigated showing a significant, but small, inhibition of the highest soluble concentration.

## Data Availability

The raw data supporting the conclusion of this article will be made available by the authors, without undue reservation.
