# Supplementary material for: Investigations of potential non-amino acid SNAT2 inhibitors
Source: Front Pharmacol. 2024 Jan 4;14:1302445. doi: 10.3389/fphar.2023.1302445 (PMC10794626; doi:10.3389/fphar.2023.1302445)
Supplement: Supplementary file 1 [file DataSheet1.docx]

**Fig. S1** ^3^H-Gly uptake (as disintegrations per minute, DPM) in hyperosmotically treated PC-3 cells in the presence or absence of 1 µM MMTC with a substrate concentration of 11.1 nM or 100 µM Gly. PC-3 cells were treated with hyperosmotic media supplemented with 200 mM raffinose 24 hours before experiments. All experiments were performed using 10 mM HEPES buffer in HBSS, pH 7.4 and the MMTC solutions contained 1% DMSO. The cells were exposed to 0.5 µCi ⋅ mL^−1 3^H-Gly at either 11.1 nM or 100 µM Gly concentrations for 5 minutes and at 37 °C. 100 µM Gly was achieved by the addition of unlabeled Gly. Values are reported as means ± SD for triplicate measurements (N=3) in a single passage of cells (n=1).


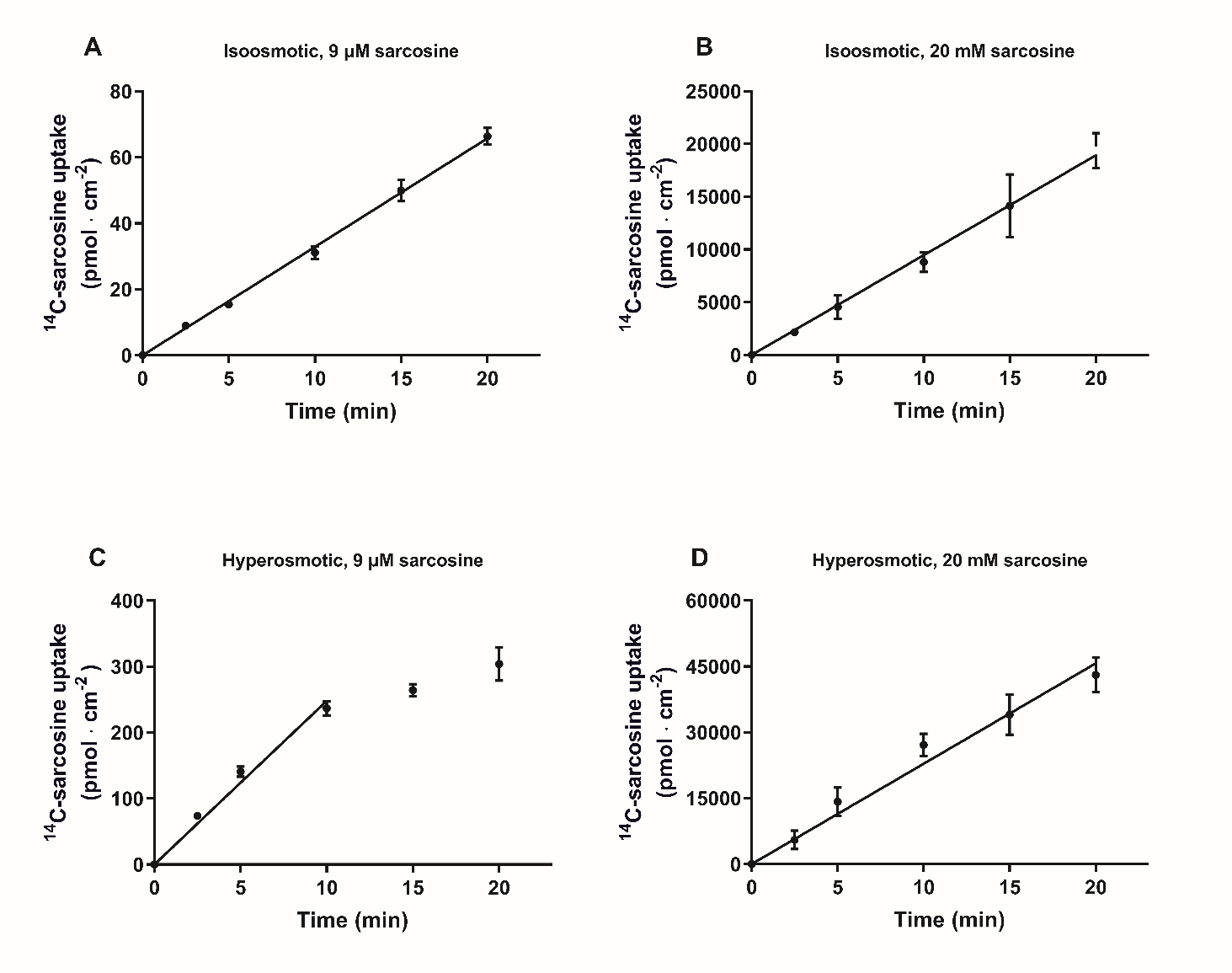


**Fig. S2:** Uptake of [^14^C]-sarcosine (0.5 µCi/mL, 9.09 µM) in PC-3 cells. All experiments were performed using 10 mM HEPES buffer in HBSS, pH 7.4 and 37°C using incubation times ranging from 2.5 to 20 minutes on a shaking table with 220 rpm. (A) Uptake in PC-3 cells cultured under isoosmotic conditions (300 mOsm/kg) with no additional unlabeled sarcosine. (Equation: y = 3.288x + 0) (B) Uptake in PC-3 cells cultured in isoosmotic conditions with 20 mM unlabeled sarcosine. (Equation: y = 946.6x + 0) (C) Uptake carried out in PC-3 cells cultured in hyperosmotic conditions (500 mOsm/kg) with no additional unlabeled sarcosine. (Equation: 24.81x + 0) (D) Experiment carried out in PC-3 cells cultured in hyperosmotic conditions with 20 mM unlabeled sarcosine. (Equation: 2285x + 0). All data are reported as mean values from experiments carried out as single experiments in three different cell passages (N = 1, n=3).

**MMTC Na^+^ adduct**

Exact mass: 491.0681 Determined mass: 491.0701

**53B H^+^ adduct**

Exact mass: 386.1975 Determined mass: 386.1961

**54F H^+^ adduct**

Exact mass: 411.0501 Determined mass: 411.0516

**55B H^+^ adduct**

Exact mass: 421.1062 Determined mass: 421.1076

**57B H^+^ adduct**

Exact mass: 444.1619 Determined mass: 444.1618

**Fig. S3** Mass spectra of compounds MMTC, 53B, 54F, 55B, and 57B obtained through electrospray ionization (ESI) in positive mode. The experimentally determined mass of the Na^+^ or H^+^ adduct of each compound is compared to the exact mass.
